# Fidelity to the ACT SMART Toolkit: an instrumental case study of implementation strategy fidelity

**DOI:** 10.1186/s43058-023-00434-2

**Published:** 2023-05-16

**Authors:** Jessica E. Tschida, Amy Drahota

**Affiliations:** 1 Department of Psychology, Michigan State University, 316 Physics Rd, East Lansing, MI 48824 USA; 2 Child and Adolescent Services Research Center (CASRC), 3665 Kearny Villa Road, Suite 200N, San Diego, CA 92123 USA

**Keywords:** Fidelity, Autism spectrum disorder, Blended implementation strategy, Implementation process strategy, Capacity-building, Case study, Implementation fidelity, Community-based setting

## Abstract

**Background:**

Evidence-based practices (EBPs) are shown to improve a variety of outcomes for autistic children. However, EBPs often are mis-implemented or not implemented in community-based settings where many autistic children receive usual care services. A blended implementation process and capacity-building implementation strategy, developed to facilitate the adoption and implementation of EBPs for autism spectrum disorder (ASD) in community-based settings, is the Autism Community Toolkit: Systems to Measure and Adopt Research-based Treatments (ACT SMART Toolkit). Based on an adapted Exploration, Adoption decision, Preparation, Implementation, Sustainment (EPIS) Framework, the multi-phased ACT SMART Toolkit is comprised of (a) implementation facilitation, (b) agency-based implementation teams, and (c) a web-based interface. In this instrumental case study, we developed and utilized a method to evaluate fidelity to the ACT SMART Toolkit. This study responds to the need for implementation strategy fidelity evaluation methods and may provide evidence supporting the use of the ACT SMART Toolkit.

**Methods:**

We used an instrumental case study approach to assess fidelity to the ACT SMART Toolkit during its pilot study with six ASD community agencies located in southern California. We assessed adherence, dose, and implementation team responsiveness for each phase and activity of the toolkit at both an aggregate and individual agency level.

**Results:**

Overall, we found that adherence, dose, and implementation team responsiveness to the ACT SMART Toolkit were high, with some variability by EPIS phase and specific activity as well as by ASD community agency. At the aggregate level, adherence and dose were rated notably lowest during the preparation phase of the toolkit, which is a more activity-intensive phase of the toolkit.

**Conclusions:**

This evaluation of fidelity to the ACT SMART Toolkit, utilizing an instrumental case study design, demonstrated the potential for the strategy to be used with fidelity in ASD community-based agencies. Findings related to the variability of implementation strategy fidelity in the present study may also inform future adaptations to the toolkit and point to broader trends of how implementation strategy fidelity may vary by content and context.

**Supplementary Information:**

The online version contains supplementary material available at 10.1186/s43058-023-00434-2.

Contributions to the literature

- Assessing implementation strategy fidelity is critical to advance the field of implementation science but is rarely evaluated in extant literature.
- In an instrumental case study, we found high but variable fidelity to a blended implementation strategy (ACT SMART Toolkit) for facilitating EBP adoption and implementation in autism community agencies.
- Findings indicate that community-based providers utilized the ACT SMART Toolkit with fidelity, supporting preliminary evidence that the toolkit may facilitate EBP implementation within community-based autism service contexts.
- The paper contributes an innovative model for assessing implementation strategy fidelity and identifies important influences of strategy content and context.

## Background

### Autism spectrum disorder

An autism spectrum disorder (ASD) affects approximately 1 in 44 children in the USA and has been identified as a public health concern estimated to cost 461 billion dollars a year for services and treatment by 2030 [1–3]. ASD is characterized by social and communication difficulties as well as restricted and repetitive behaviors and interests. Further, ASD commonly co-occurs with anxiety disorders, obsessive–compulsive disorder, attention deficit hyperactivity disorder, and/or oppositional defiant disorder [4–6]. Additionally, children on the autism spectrum have higher rates of behaviors such as self-injury, aggression, tantrums, and property destruction compared to neurotypical peers [7–9].

Both the core features and co-occurring disorders and behaviors of ASD have been found to predict unsatisfactory outcomes in quality-of-life factors. This includes peer relationships, educational attainment, employment, and independent living in adulthood [5, 10, 11]. Associations between autistic^1^ characteristics and unsatisfactory quality-of-life outcomes are also maintained by systemic barriers to the inclusion of autistic individuals. These barriers include societal stigma and lack of appropriate accommodations in education, employment, and housing opportunities [12–14].

The prevalence rate for ASD continues to grow dramatically as practices for diagnosis improve [3, 15]. However, despite their potential to improve outcomes for autistic youth and reduce individual and societal costs [16–18], barriers to community-level identification and intervention remain [3, 19]. Evidence-based practices (EBPs) have been shown to improve a variety of outcomes for autistic children. However, EBPs are often inconsistently implemented or mis-implemented in community-based settings where many autistic children receive services [20–24]. As a result, there is a considerable number of children on the autism spectrum not receiving therapeutic practices empirically demonstrated to improve outcomes as part of their usual care. Thus, there is a need to identify, develop, and evaluate strategies facilitating the adoption, implementation, and sustained use of EBPs for ASD within community settings.

### ACT SMART implementation toolkit

Drahota and colleagues [20, 25, 26] developed a packaged implementation process tool designed to facilitate autism EBP adoption, preparation, uptake, and sustained use in autism community-based agencies. The Autism Community Toolkit: Systems to Measure and Adopt Research-based Treatments (ACT SMART Toolkit) was developed through a review of existing implementation strategy taxonomies and evidence [27, 28] and by incorporating insights from collaborative community and academic partners through a community-academic partnership [29]. The ACT SMART Toolkit was developed to have steps and activities aligned with the multi-phased Exploration, Preparation, Implementation, Sustainment (EPIS) implementation framework that was adapted for this setting [20, 30, 31].

The explicit goal of the ACT SMART Toolkit during development was to co-create a systematized, yet flexible, process and accompanying set of tools that would facilitate the adoption, implementation, and sustainability of ASD EBPs within community settings [27, 30]. Specifically, the ACT SMART Toolkit is comprised of three implementation strategies: implementation facilitation, agency-based implementation teams (e.g., capacity-building implementation strategy), and a web-based interface (e.g., implementation process strategy) that provides access to the steps and activities that facilitate momentum within and between implementation phases [25–28, 30].

**Fig. 1:**
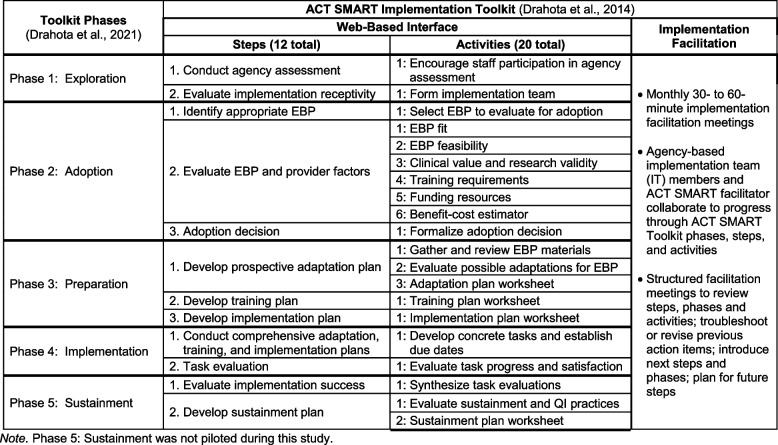
ACT SMART Implementation Toolkit steps and activities to support EBP implementation

In practice, the ACT SMART Toolkit and implementation facilitator guide ASD agency implementation teams to explore their agency’s receptivity to implementing a new EBP, identify and decide upon an EBP that meets their agency’s needs, prospectively plan to implement the EBP (e.g., adaptation, training, discrete implementation strategy use), evaluate the EBP implementation process, and develop a plan for EBP sustainment (see Fig. 1; [25]). Of note, the ACT SMART Toolkit was designed to build capacity within agencies to utilize a systematic implementation process and was developed to be used flexibly (e.g., move backward, skip activities or steps, etc.) to meet the specific needs of individual ASD agencies. That is, the ACT SMART Toolkit was designed to allow for individualization and flexibility within a structured set of phases, steps, and activities [26].

Importantly, the ACT SMART Toolkit has been pilot tested with six ASD community-based agencies. Preliminary work by Drahota and colleagues suggests that the toolkit is feasible, acceptable, and useful to agency implementation teams [Drahota A, Meza R, Martinez JI, Sridhar A, Bustos TE, Tschida J, Stahmer A, Aarons GA: Feasibility, acceptability, and utility of the ACT SMART implementation toolkit, in preparation]. In addition, Sridhar and Drahota [32] found that the toolkit facilitates clinically meaningful changes in agency provider- and supervisor-reported EBP use. Moreover, Sridhar and colleagues [33] identified salient facilitators (i.e., facilitation, facilitation meetings, and phase-specific activities) and salient barriers (i.e., website issues, perceived lack of resources, and contextual factors within ASD community agencies such as time constraints and funding) to the utilization of the ACT SMART Toolkit in the pilot study. Together, these findings suggest that the ACT SMART Toolkit may facilitate the adoption and implementation of ASD EBPs within community-based settings but likely needs revision to overcome factors that may limit its effectiveness. Appropriately powered quasi- or experimental research, necessary to test the toolkit’s effectiveness, will require the assessment and reporting of implementation strategy fidelity per the standards for reporting implementation studies [34].

### Implementation strategy fidelity

Fidelity is a construct that assesses the extent to which individuals (e.g., providers) deliver a strategy as planned [35–37]. Researchers have proposed components that contribute to fidelity should include (1) adherence to the outlined procedures, (2) proportion of the strategy received (i.e., dose), (3) extent of individual responsivity to the strategy (i.e., participant responsiveness), (4) quality of implementation, and (5) differentiation from unspecified procedures [38, 39]. Researchers have also proposed that quality and differentiation primarily capture the characteristics of an EBP being implemented whereas adherence, dose, and participant responsiveness are particularly relevant fidelity constructs for implementation strategy fidelity [37, 40].

Dusenbury [38] defines *adherence* as the extent to which activities are consistent with the way a strategy is proposed, *dose* as the amount of strategy content received by participants, and *participant responsiveness* as the extent to which participants are engaged by and involved in the strategy. In relation to the fidelity to implementation strategies, including implementation process strategies and capacity-building implementation strategies, participants could refer to agency implementation teams. Agency implementation teams are groups of individuals within an agency responsible for guiding EBP implementation [41].

Fidelity is also considered dynamic and may be influenced by factors such as provider characteristics, the setting, and/or complexity of the strategy [37, 42]. Assessing implementation strategy fidelity, especially to implementation process and blended implementation strategies, may help implementation strategy developers further understand which components of an implementation strategy may be core functions needed to produce desired outcomes and which may be adapted to account for varying contextual characteristics [43–45]. Assessing fidelity may also improve the generalizability and reproducibility of implementation strategies [46]. Of course, this is contingent upon an ability to determine whether implementation of the strategy remained consistent with its underlying theory [47, 48]. Notably, increasing understanding about how implementation strategies work has been identified as a research priority within the field of dissemination and implementation science [49–51].

Despite its importance, fidelity to implementation strategies, including implementation process and capacity-building implementation strategies [27, 46], has rarely been assessed; instead, research has often focused only on fidelity to the EBPs being implemented [37, 52]. Indeed, Slaughter et al. [37] conducted a scoping review that indicated no articles reporting fidelity to implementation strategies included definitions or conceptual frameworks for assessing implementation strategy fidelity. To our knowledge, few studies have examined fidelity to an implementation strategy and only one recent study has used a guiding theoretical framework [52, 53].

### Present study

This study utilized an instrumental case study approach to assess fidelity to the ACT SMART toolkit during its pilot study to extract insights into the use of the toolkit as well as implementation strategy fidelity methods more broadly [54]. Examining implementation strategy fidelity can provide insight into the overall potential for ASD community-based agencies to use the toolkit as planned and, if effective, ultimately implement and sustain EBP use. This information may be particularly useful for implementation practitioners using the toolkit with ASD community-based agencies in the future and needing to discern when fidelity or adaptation to toolkit activities is most appropriate. Indeed, ASD community-based agencies may have competing priorities and contextual barriers to completing the toolkit in its entirety with fidelity [33]. Further, this study provides one of the first process models to assess fidelity to a packaged implementation process tool comprised of multiple implementation strategies. This model may inform a broader understanding of implementation strategy fidelity and contribute to underlying theory. Specifically, we examined fidelity to the ACT SMART Toolkit at an aggregate and individual agency level according to adherence, dose, and participant responsiveness during its pilot study.

## Methods

### Participants

A total of six ASD community agencies located in Southern California were included in the pilot study of the ACT SMART toolkit. Four of the ASD community agencies were Applied Behavior Analysis (ABA) organizations, one was an ABA and mental health organization, and one agency was a Speech and Language Pathology organization. Participating agency leaders (*n* = 6) attended an ACT SMART Toolkit orientation meeting describing implementation science concepts and the ACT SMART Toolkit components; two of the agency leaders had been involved in the community-academic partnership that advised on the development of the Toolkit. Prior to attending an orientation for the ACT SMART Toolkit, agency leaders completed self-report measures of implementation and ACT SMART Toolkit knowledge. Rated on a 5-point Likert scale (0 = “Not at all knowledgeable” to 4 = “Extremely knowledgeable”), descriptive analyses indicate that they had moderate knowledge of implementation, generally (*Median* = 3.0), expected implementation outcomes (*Median* = 2.0), implementation barriers (*Median* = 3.0), the purpose of implementation (*Median* = 2.0), and the purpose of the ACT SMART Toolkit (*Median* = 2.0), activities (*Median* = 1.0), implementation teams (*Median* = 1.0), and facilitation meetings (*Median* = 1.0) [30].

Thereafter, agency leaders developed agency-based implementation teams composed of agency staff (Table 1 provides implementation team demographic and discipline information). At least one agency leader was required for each implementation team to ensure that adoption and implementation planning decisions could be made without additional approvals. Eligibility criteria included (1) holding the role of CEO, director, or leading decision-maker regarding treatment use at an ASD community agency eligible to participate in the ACT SMART pilot study; (2) willingness to participate in the pilot study for 1 year; and (3) agreement to provide feedback after completing each phase of the pilot study. The agency leader for each participating agency then invited up to four other agency staff members (i.e., supervisors and direct providers) to complete their agency’s implementation team. Eligibility criteria for implementation team members were to agree to complete the toolkit and provide feedback about its feasibility, acceptability, and utility.

**Table 1:** Demographic and discipline information across implementation teams

	**Agency leaders** (*n* = 7)	**Supervisors** (*n* = 8)	**Direct providers** (*n* = 1)
**Sex assigned at birth (females)**	100%	100%	100%
**Race**			
White	100%	25%	100%
Mixed race	-	25%	-
Prefer not to answer	-	12.5%	-
Missing	-	37%	-
**Education level**			
Master’s degree	42.9%	50%	100%
Doctorate	57.1%	12.5%	-
Missing	-	37%	-
**Discipline**			
Psychology	28.6%	25%	-
Behavior Specialist	28.6%	25%	100%
Speech/Language/Communication	28.6%	12.5%	-
Education	14.3%	-	-
Missing	-	37%	-

Five of the six ASD community agencies completed all phases of the ACT SMART toolkit. These agencies each chose to adopt the EBP, Video Modeling, from a menu of three EBPs (for study details, see [30]). One ABA agency chose not to adopt an EBP at the end of the adoption decision phase of the toolkit because the implementation team did not find any of the EBPs to meet the needs of the agency (e.g., lack of agency-EBP fit).

### Materials and procedure

As part of the pilot study, a research assistant served as an independent observer and evaluated implementation teams’ fidelity using the Implementation Milestones form, adapted with permission from the Stages of Implementation Completion [55], and the ACT SMART Activity Fidelity form (Drahota A, Martinez JI: ACT SMART Milestones and Activity Fidelity Forms, unpublished). The ACT SMART Implementation Milestones form required the independent observer to record a Yes or No answer (scored as 1 and 0, respectively) for whether activities during pre-implementation and phase 1 through phase 4 of the ACT SMART Toolkit were completed (see Additional file 1: Appendix A). Scores were converted into percentages to assist with interpretation. The ACT SMART Activity Fidelity form presented more detailed questions regarding completion of activities during Phase 2: Adoption; Phase 3: Preparation; and Phase 4: Implementation. The independent observer recorded a Yes or No answer (scored as 1 and 0, respectively) for whether implementation teams completed each activity and then rated the amount of the form completed using a 4-point Likert scale where 0 = “Nothing Completed”, 1 = “Minimally Completed (1–2 items)”, 2 = “Moderately Completed (3–4 items)”, and 3 = “Mostly/All Completed (5–6 items)” (see Additional file 1: Appendix B).

In addition to the observational data collected using the ACT SMART Implementation Milestones form and the ACT SMART Activity Fidelity form, ACT SMART facilitators rated implementation team engagement using the ACT SMART Implementation Team Engagement Rating Scale that was created by the toolkit developers. Immediately after each facilitation meeting, the ACT SMART facilitator(s) rated implementation team engagement in ACT SMART activities and facilitation meetings since the last facilitation meeting occurred. Engagement ratings were completed using a 5-point Likert scale where 1 = “Not at all engaged”, 2 = “Slightly Engaged”, 3 = “Moderately Engaged”, 4 = “Very Engaged”, and 5 = “Extremely Engaged” (see Additional file 1: Appendix C).

In the present study, we used the operational definitions from Dusenbury [38] and an overall scoring rubric for implementation strategy fidelity developed by Slaughter et al. [37] as the basis for using the ACT SMART Implementation Milestones form, ACT SMART Activity Fidelity form, and ACT SMART Implementation Team Engagement Rating Scale to assess implementation strategy fidelity via adherence, dose, and participant responsiveness, respectively.

### Analysis plan

We used an instrumental case study approach to explore both fidelity to the ACT SMART Toolkit and potential generalizations to a broader underlying theory of implementation strategy fidelity. The Standards for Reporting Implementation Studies (StaRI) checklist was used to assist reporting, given that the ACT SMART Toolkit is a packaged, blended implementation process tool developed to increase EBP use in ASD community agencies [Drahota A, Meza R, Martinez JI, Sridhar A, Bustos TE, Tschida J, Stahmer A, Aarons GA: Feasibility, acceptability, and utility of the ACT SMART implementation toolkit, in preparation]. First, we assessed adherence, dose, and participant responsiveness for the ACT SMART Toolkit overall as well as for each phase and activity of the toolkit. Utilizing the ACT SMART Implementation Milestones form, we assessed *adherence* via a Yes/No answer to whether implementation milestones were completed. Overall, by phase, and by activity, we calculated the average percentage of “Yes” answers for required toolkit activities. We assessed *dose* by analyzing Likert scales on the ACT SMART Activity Fidelity form evaluating how much of each activity was completed. Overall, by phase, and by activity, we calculated the median dose rating. Finally, we assessed *participant responsiveness* by analyzing the Likert scales on the ACT SMART Implementation Team Engagement Rating Scale and used dates of completion to confirm phase. Overall and by phase, we calculated the median participant responsiveness rating. We did not calculate the median participant responsiveness rating by activity as ratings for engagement were only given by phase. We also calculated an average percent agreement on participant responsiveness ratings from facilitation meetings in which multiple facilitators were present. All facilitators attended informal trainings on rating participant responsiveness using the ACT SMART Implementation Team Engagement Scale. During supervision sessions with facilitators, facilitators engaged in discussions about their rationale for participant responsiveness ratings for each facilitation meeting. Lastly, we calculated overall, phase, and activity adherence, dose, and participant responsiveness for each agency implementation team.

## Results

### Aggregate fidelity to the ACT SMART Toolkit

Agency implementation teams adhered to an overall average of 90% (*SD* = 11.3%) of ACT SMART Toolkit activities. Average adherence ranged from 74% (*SD* = 19.5%) completion of toolkit activities during the preparation phase of the toolkit to 100% (*SD* = 0%) completion of toolkit activities during the implementation phase of the toolkit (see Table 2). While the completion rate for individual activities within phases was also relatively high across agencies, there was some variability.

**Table 2:** Adherence to the ACT SMART Implementation Toolkit in aggregate and by individual agency implementation team

	**Aggregate** *M%* (*SD*)	**Agency 1** *M%* (*SD*)	**Agency 2** *M%* (*SD*)	**Agency 3**^***a***^ *M%* (*SD*)	**Agency 4** *M%* (*SD*)	**Agency 5** *M%* (*SD*)	**Agency 6** *M%* (*SD*)
**Overall adherence scores**	**90% (11.3)**	**90.8% (14.5)**	**93.3% (14.9)**	**91.7% (14.4)**	**89.5% (17.4)**	**88.0% (26.8)**	**85.3% (20.2)**
**Pre-implementation**	**100%**	**100%**	**100%**	**100%**	**100%**	**100%**	**100%**
Agency first contacted	100%	100	100	100	100	100	100
Agency interest indicated	100%	100	100	100	100	100	100
Agency recruitment meeting	100%	100	100	100	100	100	100
Orientation meeting attendance	100%	100	100	100	100	100	100
**Phase 1: Exploration**	**83% (18.0)**	**66.7% (57.7)**	**66.7% (57.7)**	**100%**	**100%**	**100%**	**66.7% (57.7)**
Recruit for agency assessment	83% (40.8)	100	0	100	100	100	100
Agency assessment link sent	100%	100	100	100	100	100	100
Staff response rate ≥ 75%	67% (51.6)	0	100	100	100	100	0
**Phase 2: Adoption**	**92% (17.8)**	**87.5% (35.4)**	**100%**	**75.0% (46.3)**	**87.5% (35.4)**	**100%**	**100%**
Treatment selection	100%	100	100	100	100	100	100
Evaluate fit	100%	100	100	100	100	100	100
Evaluate feasibility	100%	100	100	100	100	100	100
Evaluate clinical utility and validity	83% (40.8)	100	100	0	100	100	100
Evaluate training requirements	100%	100	100	100	100	100	100
Evaluate funding source	100%	100	100	100	100	100	100
Evaluate benefits and costs	50% (54.8)	0	100	0	0	100	100
Validate adoption decision	100%	100	100	100^*a*^	100	100	100
**Phase 3: Preparation**	**74% (19.5)**	**100%**	**100%**		**60% (54.8)**	**40% (54.8)**	**60% (54.8)**
Gather and review treatment materials	60% (54.8)	100	100		0	0	100
Evaluate prospective adaptations	80% (44.7)	100	100		100	0	100
Develop adaptation plan	50% (70.7)	100	N/A		N/A	0	N/A
Develop training plan	100%	100	100		100	100	100
Develop implementation plan	80% (44.7)	100	100		100	100	0
**Phase 4: Implementation**	**100%**	**100%**	**100%**		**100%**	**100%**	**100%**
Carry out adaptation plan	100%	100	N/A		N/A	N/A	N/A
Carry out training plan	100%	100	100		100	100	100
Carry out implementation plan	100%	100	100		100	100	100^*b*^

Adherence scoring range is 0–100%. M% = Mean percentage. *N* = 6 implementation teams

^a^Agency implementation team made the decision to not adopt an EBP

^b^Agency implementation team created and carried out an implementation plan in Phase 4

Related to dose, the independent observer gave agency implementation teams an overall median rating of “Mostly/All Completed” (*Median* = *3.0*). The lowest median dose rating was between “Moderately Completed” to “Mostly/All Completed” (*Median* = 2.5) during the preparation phase whereas the highest median dose ratings were “Mostly/All Completed” (*Median* = 3.0) during the adoption and implementation phases of the toolkit (see Table 3). Consistent with observations of adherence, there were lower dose ratings for activities such as the benefit–cost estimator, gathering treatment materials, and developing adaptation and implementation plans compared to higher completion rates for activities related to treatment evaluation, funding, training, and carrying out developed plans. Here, it should be noted that dose ratings by activity could not be calculated for the implementation phase given that evaluation surveys during this phase were designed to be dynamic and capture completion of individualized sets of tasks by agency [Drahota A, Meza R, Martinez JI, Sridhar A, Bustos TE, Tschida J, Stahmer A, Aarons GA: Feasibility, acceptability, and utility of the ACT SMART implementation toolkit, in preparation].

**Table 3:** Dose to the ACT SMART Implementation Toolkit in aggregate and by individual agency implementation team

	**Aggregate** *Median*	**Agency 1** *Median*	**Agency 2** *Median*	**Agency 3**^***a***^ *Median*	**Agency 4** *Median*	**Agency 5** *Median*	**Agency 6** *Median*
**Overall dose scores**	**3.0**	**3.0**	**3.0**	**1.5**	**3.0**	**3.0**	**3.0**
**Phase 1: Exploration**	†	†	†	†	†	†	†
**Phase 2: Adoption**	**3.0**	**3.0**	**3.0**	**1.5**	**3.0**	**3.0**	**3.0**
Evaluate fit	3.0	3	3	3	3	3	3
Evaluate feasibility	3.0	3	3	3	3	3	3
Evaluate clinical utility and validity	3.0	3	3	0	3	3	3
Evaluate training requirements	3.0	*	3	*	2	*	3
Evaluate funding source	3.0	3	1	3	3	3	1
Evaluate benefits and costs	1.0	-	3	0	0	3	1
Validate adoption decision	3.0	3	3	0^*a*^	3	3	0
**Phase 3: Preparation**	**2.5**	**3.0**	**2.5**		**2.5**	**0**	**2.0**
Gather and review treatment materials	1.0	1	1		0	0	1
Evaluate prospective adaptations	3.0	3	3		3	0	3
Develop adaptation plan	1.5	3	N/A		N/A	0	N/A
Develop training plan	3.0	3	3		3	1	3
Develop implementation plan	2.0	1	2		2	3	0
**Phase 4: Implementation **^***b***^	**3.0**	**3.0**	**3.0**		**3.0**	**3.0**	**3.0**

Dose scoring ranges from 0 (Nothing completed) to 3 (Mostly/All completed [5–6 items]). *N* = 6 implementation teams

^a^Agency implementation team made the decision to not adopt an EBP, therefore, did not progress past Phase 2

^b^Dose scoring for phase 4: implementation from responses to amount completion for implementation evaluation surveys (see Additional file 1: Appendix B)

^†^Denotes that this is not applicable for the fidelity domain

^*^Agency implementation team indicated that there were no training requirements while completing form

- indicates missing data

For participant responsiveness, ACT SMART facilitators rated agency implementation teams with a median rating falling between “Moderately Engaged” and “Very Engaged” (*Median* = *3*.8). The lowest median participant responsiveness rating was between “Moderately Engaged” and “Very Engaged” (*Median* = *3.5*) during the adoption decision phase of the toolkit. The highest median participant responsiveness rating was at “Extremely Engaged” (*Median* = 5.0) during the implementation phase (see Table 4). For facilitation meetings with multiple ACT SMART facilitators present, there was a 92.43% average agreement on participant responsiveness ratings.

**Table 4:** Participant responsiveness to the ACT SMART Implementation Toolkit in aggregate and by individual agency implementation team

	**Aggregate** *Median*	**Agency 1** *Median*	**Agency 2** *Median*	**Agency 3**^***a***^ *Median*	**Agency 4** *Median*	**Agency 5** *Median*	**Agency 6** *Median*
**Overall Participant Responsiveness Scores**	**3.8**	**3.8**	**4.8**	**3.1**	**3.8**	**4.3**	**3.8**
Phase 1: Exploration	3.8	4	4	3.25	3.5	5	3
Phase 2: Adoption	3.5	3.5	5	3.0^*a*^	3.5	4.5	3.5
Phase 3: Preparation	4.0	3.5	4.5		4.0	4.0	4.0
Phase 4: Implementation	5.0	5	5		4.0	4.0	5

Participant responsiveness scoring ranges from 1 (Not at all engaged) to 5 (Extremely engaged). *N* = 6 implementation teams

^a^Agency implementation team made the decision to not adopt an EBP, therefore, did not progress past Phase 2

### Individual agency fidelity to the ACT SMART Toolkit

Across agencies, there was generally high adherence to toolkit activities; the lowest agency implementation team adhered to an overall average of 85.3% (*SD* = 20.2%) of toolkit activities (Table 2). While there was some variability in adherence across phases and activities by agency, there was no readily identifiable pattern of agencies consistently having lower or higher adherence compared to other agencies. Consistent with other results, the preparation phase appeared to have the lowest adherence ratings across agencies.

Agencies also all had generally high dose ratings for toolkit activities, except for the one agency (Agency 3) that chose not to adopt an EBP at the end of Phase 2: Adoption (Table 3). Like the ratings of adherence by agency, there was variability in dose ratings but no consistent identifiable patterns. Further, the preparation phase had the lowest dose ratings across agencies.

Consistent with both observations of adherence and dose ratings across agencies, all agencies also had relatively high ratings of participant responsiveness (Table 4). The agency with the lowest median participant responsiveness rating was rated between “Moderately Engaged” to “Very Engaged” (*Median* = 3.1). However, in contrast to observations of adherence and dose ratings, agencies did not appear to have lower participant responsiveness during the preparation phase compared to other toolkit phases.

## Discussion

### Fidelity to the ACT SMART Toolkit

Our investigation used an instrumental case study approach to evaluate implementation strategy fidelity to the ACT SMART Toolkit by assessing observational descriptive ratings of adherence, dose, and participant responsiveness. Our evaluation provides one of the first models of assessing fidelity to a blended implementation process and capacity-building implementation strategy. In addition, our evaluation provides important insights into both the potential for ASD community-based agencies to use the toolkit effectively and implementation strategy fidelity more broadly. Overall, we found that adherence, dose, and participant responsiveness to the ACT SMART Toolkit were relatively high, which supports the potential for the toolkit to be used with fidelity in ASD community agencies. Despite their potential to improve outcomes for a growing clinical population, EBPs for ASD are often inconsistently or mis-implemented in community settings. Thus, understanding the effective use of implementation strategies, such as the ACT SMART Toolkit, could contribute to reducing the EBP research-to-practice gap [20–24].

Although we found fidelity to be high overall, there was some variability in implementation strategy fidelity by toolkit phase. Specifically, we found that adherence and dose were rated the lowest in the preparation phase (Phase 3) at an aggregate level. However, we were underpowered to determine whether the differences by phase were statistically significant. One possible rationale for the descriptive finding of lower adherence and dose in Phase 3 is that there were substantial differences in the cognitive or informational demands of toolkit activities by phase. Indeed, the preparation phase required gathering materials, evaluating prospective adaptations, and developing training and adaptation plans whereas the implementation phase required carrying out and evaluating the developed plans. Notably, there were both lower adherence and dose ratings for toolkit activities such as developing adaptation and implementation plans compared to toolkit activities related to evaluating treatments, funding, and training. Thus, the lower adherence and dose in the preparation phase may reflect the need to reduce the amount or intensity of toolkit activities to better align with ASD community agencies’ capacity to plan for implementation. Considering recently identified context-specific barriers and facilitators to the ACT SMART Toolkit, such as availability of funding, time, and staffing, would also likely be critical to enhancing the toolkit overall [33, 56].

Another potential rationale for lower adherence and dose during the preparation phase may be that ASD community agencies perceived greater value in implementing the chosen EBP than in planning for its implementation. While agency implementation teams were rated as moderately to very engaged during the preparation phase, it is unclear how well facilitators were able to emphasize the important relationship between planning and implementation. However, researchers have recently proposed that fostering this understanding is necessary to support successful and sustainable implementation [57]. Thus, the ACT SMART Toolkit may also benefit from incorporating a greater focus on the practical importance of planning for implementation of EBPs.

### Implementation strategy fidelity theory

Our instrumental case study assessment of fidelity to the ACT SMART Toolkit within ASD community agencies notably provides one of the first process models of assessing blended implementation strategy fidelity. Although a considerable amount of research has been conducted on *intervention fidelity*, few researchers have explored *implementation strategy fidelity* [37, 52, 53]. For example, Slaughter et al. [37] found that no studies reporting on fidelity to implementation included a specific definition or theoretical framework for assessing implementation strategy fidelity. To our knowledge, only Berry and colleagues [52] recently adapted the Conceptual Framework for Implementation Fidelity to guide their evaluation of fidelity to practice facilitation as a strategy to improve primary care practices’ adoption of evidence-based guidelines for cardiovascular disease.

Despite limited research, evaluating and understanding implementation strategy fidelity have important implications and are identified as research priorities within dissemination and implementation science [47–51]. High fidelity to an implementation strategy may be reflective of other important implementation outcomes, such as high acceptability, appropriateness, and feasibility [58, 59]. Further, implementation strategy fidelity may inform the determination of which components of a strategy are required to produce change (e.g., core components) and which can be removed or adapted to account for varying contextual characteristics [43–45]. This knowledge may allow for demand optimization when the implementation strategy is being used, which may be particularly important when users of an implementation strategy have competing priorities or contextual factors that make completing the entirety of a blended implementation strategy difficult [33].

From our instrumental case study of ACT SMART Toolkit fidelity, we have demonstrated that fidelity to blended implementation strategies, including implementation planning strategies and capacity-building strategies, is possible. Further, implementation strategy fidelity may vary according to differing components of a strategy, such as components focusing on preparation for implementation versus components focusing on implementation itself. We also observed that implementation strategy fidelity may vary by context. Here, implementation strategy fidelity was observed to vary across different ASD community agencies using the ACT SMART Toolkit. These findings suggest that a next step to further understand implementation strategy fidelity may be investigating shifts across both strategy content and context. Importantly, increasing this understanding could then also inform commonly needed adaptations to improve implementation strategy fidelity.

### Strengths

We propose that the main strength of our investigation is that we demonstrate one of the first instrumental case studies to consider fidelity to a blended implementation strategy. Importantly, our assessment of fidelity to the ACT SMART Toolkit may be able to provide a framework for other evaluations of implementation strategy fidelity and inform the underlying theory of implementation strategy fidelity. Within our evaluation, we also importantly found overall high fidelity to the toolkit within ASD community-based agencies and identified potential ways in which to optimize demands of the toolkit and increase sustainability. Understanding fidelity to the toolkit within a pilot study is a critical first step before broader use with many agencies in appropriately powered studies.

### Limitations

In contrast, important limitations of our investigation include potential issues with measurement of specific implementation strategy fidelity variables. For example, Berry and colleagues [52] recently considered participant responsiveness as a moderator of implementation strategy fidelity rather than a component of fidelity itself as considered in our analysis. Moreover, the potential issues with measurement may have been compounded by the fact that standard measures were not used for dose and participant responsiveness. However, as an emerging field, implementation science often faces issues related to measurement and standardized measures specific to implementation strategy fidelity have not yet been developed [49, 50, 60]. Researchers have developed some standard measures for intervention fidelity, and these may be able to be adapted to assess implementation strategy fidelity in the future [61].

Another potential limitation in our investigation is that there were different raters for adherence, dose, and participant responsiveness. While an independent observer rated adherence and dose for each implementation team, participant responsiveness was rated by a facilitator following implementation teams’ facilitation meetings. Although this presents potential for bias, direct observation by independent observers and even implementers has been found to be more accurate than collecting reports directly from participants [61]. Further, when two facilitators independently gave ratings for participant responsiveness, there were high rates of agreement. Ratings were also only given for implementation teams as one unit rather than individually for each implementation team member. In the present study, rating at the level of implementation teams was practical given that ASD community agencies may face high rates of staff turnover [33]. However, future research would benefit from examining whether implementation strategy fidelity varies by implementation team member or staff role.

Moreover, while we were generally able to assess implementation strategy fidelity by toolkit phase and activities, we were unable to assess all variables for all activities and by toolkit facet (i.e., web-based interface versus facilitation meetings). Thus, we are unable to make conclusions about all activities and the impact of the blended nature of the toolkit on implementation strategy fidelity. Further, our results may not generalize to discrete implementation strategies, which may benefit from their own instrumental case studies.

Lastly, the most important limitation of our assessment of fidelity to the ACT SMART Toolkit was the limited sample size that rendered us under-powered to fully evaluate relationships between implementation strategy fidelity and EBP use. Moreover, in the limited sample, implementation teams that completed each phase of the toolkit all chose to adopt video modeling. While this may reflect the particular ease of adopting video modeling (e.g., low training requirements and cost), it is unclear whether results would vary with a different choice of EBP. Our limited sample size also precluded us from considering additional factors such as implementation team and provider demographics and organizational climate within ASD community agencies. While we were able to observe variable implementation strategy fidelity across ASD community agencies, we were not yet able to identify consistent patterns related to higher or lower implementation strategy fidelity. However, there is evidence that some of these factors may moderate the relationship between implementation strategy fidelity to the ACT SMART toolkit and increased EBP use [62].

Future research would benefit from consideration of potential moderators of implementation strategy fidelity and utilizing standard measures and independent raters [60–65]. In addition, future studies may benefit from a design intended to systematically evaluate fidelity to all components of a strategy. These lines of research may provide further insight into both effective use of the ACT SMART Toolkit and advancing the field of implementation science more broadly.

## Conclusions

By utilizing an instrumental case study approach, we advanced understanding of effective use of the ACT SMART Toolkit as well as the theory of implementation strategy fidelity more broadly. We found that the ACT SMART Toolkit has the potential to be used with high fidelity in ASD community-based agencies. However, we also found that there was some variability in fidelity among toolkit phases, which points to possible adaptations needed to improve toolkit use even further. Considering adaptations may be critical as these findings may reflect that fidelity to blended implementation strategies is dynamic and affected by both strategy content and context. By increasing the use of and fidelity to effective implementation strategies that facilitate EBP adoption, utilization, and sustainment within community-based settings, there is potential to increase overall public health.

## Supplementary Information

**Additional file 1. Appendix A. **ACT SMART Implementation Milestones Form. **Appendix B.** ACT SMART Activity Fidelity Form. **Appendix C.** ACT SMART Implementation Team Engagement Rating Scale.

## Data Availability

The datasets used and/or analyzed during the current study are available from the senior author (AD) upon reasonable request.

## Abbreviations

ASD: Autism spectrum disorder
EBP: Evidence-based practice
ACT SMART Toolkit: Autism Community Toolkit: Systems to Measure and Adopt Research-Based Treatments
EPIS model: Exploration, Preparation, Implementation, Sustainment model
ABA: Applied Behavior Analysis

## Footnotes

1 We use “identity-first” language in some instances due to recent studies indicating that identity-first language is preferred by some autistic individuals and a recent review highlighting potentially ableist language in autism research [12].
