# Supplementary material for: Fidelity to the ACT SMART Toolkit: an instrumental case study of implementation strategy fidelity
Source: Implement Sci Commun. 2023 May 16;4:52. doi: 10.1186/s43058-023-00434-2 (PMC10189967; doi:10.1186/s43058-023-00434-2)
Supplement: Supplementary file 1 — Additional file 1. Appendix A. ACT SMART Implementation Milestones Form. Appendix B. ACT SMART Activity Fidelity Form. Appendix C. ACT SMART Implementation Team Engagement Rating Scale. [file 43058_2023_434_MOESM1_ESM.pdf]

Supplemental Material

Appendix A. ACT SMART Implementation Milestones Form

Phase	ACT SMART Phase	Activity	Date Initiated	Completed (Yes/No)	Date Completed
Pre-Implementation	Recruitment	Agency first contacted			
		Agency interest indicated			
		Agency recruitment meeting			
		Orientation workshop			
Implementation	Phase 1<br>Date initiated:	Meeting at agency to recruit for agency assessment			
		Emails sent to agency staff for agency assessment			
		ACT SMART agency assessment (75% staff response rate)			
	Phase 2<br>Date initiated:	Treatment selection (Phase 2, Step 1, Activity 1)			
		Evaluate treatment fit (Phase 2, Step 2, Activity 1)			
		Evaluate treatment feasibility (Phase 2, Step 2, Activity 2)			
		Evaluate clinical value and research validity (Phase 2, Step 2, Activity 3)			
		Evaluate training requirements (Phase 2, Step 2, Activity 4)			
		Evaluate funding source (Phase 2, Step 2, Activity 5)			
		Evaluate benefit-cost estimator (Phase 2, Step 2, Activity 6)			
		Make an adoption decision (Phase 2, Step 3, Activity 1)			
	Phase 3<br>Date initiated:	Gather treatment materials (Phase 3, Step 1, Activity 1)			
		Evaluate prospective treatment adaptations (Phase 3, Step 1, Activity 2)			
		Develop an adaptation plan (Phase 3, Step 1, Activity 3)			
		Develop a training plan (Phase 3, Step 2, Activity 1)			
		Develop an implementation and sustainment plan (Phase 3, Step 3, Activity 1)			
	Phase 4<br>Date initiated:	Carry out adaptation plan (Phase 4, Step 1, Activity 1)			
		Carry out training plan (Phase 4, Step 2, Activity 1)			
		Carry out implementation and sustainment plan (Phase 4, Step 3, Activity 1)			

Appendix B. ACT SMART Activity Fidelity Form

Phase 2: Treatment Selection and Adoption Decision				
**Treatment Fit (6 items; 1 area)** Phase 2, Step 2, Activity 1				
a. Was the form completed?			Yes(1)	No(0)
b. How much of the worksheet was completed?				
	0<br>Nothing Completed	1<br>Minimally Completed<br>(1-2 items)	2<br>Moderately Completed<br>(3-4 items)	3<br>Mostly/All Completed<br>(5-6 items)
**Treatment Feasibility (6 items; 1 area)** Phase 2, Step 2, Activity 2				
a. Was the form completed?			Yes(1)	No(0)
b. How much of the worksheet was completed?				
	0<br>Nothing Completed	1<br>Minimally Completed<br>(1-2 items)	2<br>Moderately Completed<br>(3-4 items)	3<br>Mostly/All Completed<br>(5-6 items)
**Clinical Value and Research Validity (10 items; 2 areas)** Phase 2, Step 2, Activity 3				
a. Was the form completed?			Yes(1)	No(0)
b. How much of the worksheet was completed?				
	0<br>Nothing Completed	1<br>Minimally Completed<br>(1-3 items)	2<br>Moderately Completed<br>(4-7 items)	3<br>Mostly/All Completed<br>(8-10 items)
c. How many areas were attended to?				
	0<br>None	1<br>Some<br>(1 area)		2<br>All<br>(2 areas)
**Training Requirements (25 items; 9 areas)** Phase 2, Step 2, Activity 4				
a. Was the form completed?			Yes(1)	No(0)
b. How much of the worksheet was completed? If no training requirements identified, circle N/A here				
	0<br>Nothing Completed	1<br>Minimally Completed<br>(1-10 items)	2<br>Moderately Completed<br>(11-19 items)	3<br>Mostly/All Completed<br>(20-25 items)
c. How many areas were attended to? If no training requirements identified, circle N/A here				
	0<br>None	1<br>Minimal<br>(1-3 areas)	2<br>Some<br>(4-7 areas)	3<br>Mostly or All<br>(8-9 areas)
**Funding Source (1-3 items; 1 area)** Phase 2, Step 2, Activity 5				
a. Was the form completed?			Yes(1)	No(0)
b. How much of the worksheet was completed? *Use judgment if fewer sections were completed*				
	0<br>Nothing Completed	1<br>Minimally Completed<br>(e.g., 1 item)	2<br>Moderately Completed<br>(e.g., 2 items)	3<br>Mostly/All Completed<br>(e.g., 3 items)
**Benefit-Cost Estimator (46 items; 7 areas)** Phase 2, Step 2, Activity 6				
a. Was the form completed?			Yes(1)	No(0)
b. How much of the worksheet was completed?				
	0<br>Nothing Completed	1<br>Minimally Completed	2<br>Moderately Completed	3<br>Mostly/All Completed
c. How many areas were attended to?				
	0<br>None	1<br>Minimal<br>(1-2 areas)	2<br>Some<br>(3-5 areas)	3<br>Mostly or All<br>(6-7 areas)
**Adoption Decision (7 items, 2 areas)** Phase 2, Step 3				
a. Was the form completed?			Yes(1)	No(0)
b. How much of the worksheet was completed?				
	0	1	2	3

Nothing Completed	Minimally Completed (1 items)	Moderately Completed (2-3 items)	Mostly/All Completed (4-5 items)

c. How many areas were attended to?

0	1	2
None	Some (1 area)	All (2 areas)

Phase 3: Planning for Implementation

Gathering Materials (1 items; 0 areas) Phase 3, Step 1, Activity 1

a. Was the form completed? Yes(1) No(0)

b. How much of the worksheet was completed?

0	1
Nothing Completed	All Completed (1 item)

Evaluating Prospective Adaptations (17 items; 2 areas) Phase 3, Step 1, Activity 2

a. Was the form completed? Yes(1) No(0)

b. How much of the worksheet was completed?

0	1	2	3
Nothing Completed	Minimally Completed (1-5 items)	Moderately Completed (6-12 items)	Mostly/All Completed (13-17 items)

c. How many areas were attended to?

0	2	3
None	Some (1 areas)	All (2 areas)

Adaptation Plan (Variable items; 5 areas) Phase 3, Step 1, Activity 3

a. Was the form completed? Yes(1) No(0) N/A (2)

b. How many areas were attended to?

0	1	2	3
None	Minimal (1 area)	Some (2-3 areas)	Mostly or All (4-5 areas)

c. How detailed were the Agency Leader/Team's comments, when made?

0	1	2	3
No Comments	Minimal Detail	Some Detail	Very Detailed

Training Plan (Variable Items; 7 areas) Phase 3, Step 2, Activity 1

a. Was the form completed? Yes(1) No(0)

b. How many areas were attended to?

0	1	2	3
None	Minimal (1-2 areas)	Some (3-5 areas)	Mostly or All (6-7 areas)

c. How detailed were the Agency Leader/Team's comments, when made?

0	1	2	3
No Comments	Minimal Detail	Some Detail	Very Detailed

Implementation and Sustainment Plan (5 areas) Phase 3, Step 3, Activity 1

a. Was the form completed? Yes(1) No(0)

b. How detailed were the Agency Leader/Team's comments, when made?

0	1	2	3
No Comments	Minimal Detail	Some Detail	Very Detailed

Phase 4: Implementation

Evaluation Survey 1

a. Was the form completed? Yes(1) No(0)

b. How much of the worksheet was completed?			
0	1	2	3
Nothing Completed	Minimally Completed	Moderately Completed	Mostly/All Completed
**Evaluation Survey 2**			
a. Was the form completed?		Yes(1)	No(0)
b. How much of the worksheet was completed?			
0	1	2	3
Nothing Completed	Minimally Completed	Moderately Completed	Mostly/All Completed
**Evaluation Survey 3**			
a. Was the form completed?		Yes(1)	No(0)
b. How much of the worksheet was completed?			
0	1	2	3
Nothing Completed	Minimally Completed	Moderately Completed	Mostly/All Completed
**Evaluation Survey 4**			
a. Was the form completed?		Yes(1)	No(0)
b. How much of the worksheet was completed?			
0	1	2	3
Nothing Completed	Minimally Completed	Moderately Completed	Mostly/All Completed
**Evaluation Survey 5**			
a. Was the form completed?		Yes(1)	No(0)
b. How much of the worksheet was completed?			
0	1	2	3
Nothing Completed	Minimally Completed	Moderately Completed	Mostly/All Completed
**Evaluation Survey 6**			
a. Was the form completed?		Yes(1)	No(0)
b. How much of the worksheet was completed?			
0	1	2	3
Nothing Completed	Minimally Completed	Moderately Completed	Mostly/All Completed
**Evaluation Survey 7**			
a. Was the form completed?		Yes(1)	No(0)
b. How much of the worksheet was completed?			
0	1	2	3
Nothing Completed	Minimally Completed	Moderately Completed	Mostly/All Completed
**Evaluation Survey 8**			
a. Was the form completed?		Yes(1)	No(0)
b. How much of the worksheet was completed?			
0	1	2	3
Nothing Completed	Minimally Completed	Moderately Completed	Mostly/All Completed
**Evaluation Survey 9**			
a. Was the form completed?		Yes(1)	No(0)

b. How much of the worksheet was completed?			
0<br>Nothing Completed	1<br>Minimally Completed	2<br>Moderately Completed	3<br>Mostly/All Completed
**Evaluation Survey 10**			
a. Was the form completed?		Yes(1)	No(0)
b. How much of the worksheet was completed?			
0<br>Nothing Completed	1<br>Minimally Completed	2<br>Moderately Completed	3<br>Mostly/All Completed
**Evaluation Survey 11**			
a. Was the form completed?		Yes(1)	No(0)
b. How much of the worksheet was completed?			
0<br>Nothing Completed	1<br>Minimally Completed	2<br>Moderately Completed	3<br>Mostly/All Completed
**Evaluation Survey 12**			
a. Was the form completed?		Yes(1)	No(0)
b. How much of the worksheet was completed?			
0<br>Nothing Completed	1<br>Minimally Completed	2<br>Moderately Completed	3<br>Mostly/All Completed
**Evaluation Survey 13**			
a. Was the form completed?		Yes(1)	No(0)
b. How much of the worksheet was completed?			
0<br>Nothing Completed	1<br>Minimally Completed	2<br>Moderately Completed	3<br>Mostly/All Completed
**Evaluation Survey 14**			
a. Was the form completed?		Yes(1)	No(0)
b. How much of the worksheet was completed?			
0<br>Nothing Completed	1<br>Minimally Completed	2<br>Moderately Completed	3<br>Mostly/All Completed
**Evaluation Survey 15**			
a. Was the form completed?		Yes(1)	No(0)
b. How much of the worksheet was completed?			
0<br>Nothing Completed	1<br>Minimally Completed	2<br>Moderately Completed	3<br>Mostly/All Completed
**Evaluation Survey 16**			
a. Was the form completed?		Yes(1)	No(0)
b. How much of the worksheet was completed?			
0<br>Nothing Completed	1<br>Minimally Completed	2<br>Moderately Completed	3<br>Mostly/All Completed
**Evaluation Survey 17**			
a. Was the form completed?		Yes(1)	No(0)

b. How much of the worksheet was completed?			
0<br>Nothing Completed	1<br>Minimally Completed	2<br>Moderately Completed	3<br>Mostly/All Completed
**Evaluation Survey 18**			
a. Was the form completed?		Yes(1)	No(0)
b. How much of the worksheet was completed?			
0<br>Nothing Completed	1<br>Minimally Completed	2<br>Moderately Completed	3<br>Mostly/All Completed
**Evaluation Survey 19**			
a. Was the form completed?		Yes(1)	No(0)
b. How much of the worksheet was completed?			
0<br>Nothing Completed	1<br>Minimally Completed	2<br>Moderately Completed	3<br>Mostly/All Completed
**Evaluation Survey 20**			
a. Was the form completed?		Yes(1)	No(0)
b. How much of the worksheet was completed?			
0<br>Nothing Completed	1<br>Minimally Completed	2<br>Moderately Completed	3<br>Mostly/All Completed

Appendix C. ACT SMART Implementation Team Engagement Rating Scale

---

ACT SMART Facilitation Meeting

Implementation Team Engagement Rating Scale – Facilitator Report

Jonathan I. Martinez, Ph.D.

Provide the following engagement ratings for the period of time from last facilitation meeting to the current facilitation meeting:

5 Extremely Engaged

- The implementation team displays great willingness to discuss progress on the ACT SMART toolkit and upcoming goals with the facilitator (i.e., team fully initiates discussion topics and/or appears fully open to discussing progress and goals).
- The implementation team contributes detailed information to identify meeting agenda topics; there is a sense of true collaboration with the facilitator
- The implementation team is extremely willing and capable of implementing the ACT SMART toolkit based on the phase they are in and topics from facilitation meeting

4 Very Engaged

- The implementation team displays much willingness to discuss progress on the ACT SMART toolkit and upcoming goals with the facilitator (i.e., team mostly initiates discussion topics and/or appears mostly open to discussing progress and goals)
- The implementation team contributes much information to identify meeting agenda topics; there is a sense of collaboration with the facilitator
- The implementation team is very willing and capable of implementing the ACT SMART toolkit based on the phase they are in and topics from facilitation meeting

3 Moderately Engaged

- The implementation team displays some willingness to discuss progress on the ACT SMART toolkit and upcoming goals with the facilitator (i.e., team is responsive to discussion topics and appears somewhat open to discussing progress and goals).
- The implementation team contributes adequate information to identify meeting agenda topics; there is a sense of consultation with the facilitator rather than collaboration.
- The implementation team is somewhat willing and capable of implementing the ACT SMART toolkit based on the phase they are in and topics from facilitation meeting

2 Slightly Engaged

- The implementation team displays minimal willingness to discuss progress on the ACT SMART toolkit and upcoming goals with the facilitator (i.e., team is minimally responsive to discussion topics and appears minimally open to discussing progress and goals).
- The implementation team contributes minimally to identifying meeting agenda topics; there is a sense of indifference with facilitation meetings
- The implementation team appears indifferent and minimally capable of implementing the ACT SMART toolkit based on the phase they are in and topics from facilitation meeting

1 Not at all engaged

- The implementation team is not willing to discuss progress on the ACT SMART toolkit and upcoming goals with the facilitator (i.e., team is not responsive to discussion topics and not open to discussing progress and goals).
- The implementation team does not contribute to identifying meeting agenda topics; there is a sense of not wanting to participate in facilitation meetings.
- The implementation team is not willing and capable of implementing the ACT SMART toolkit based on the phase they are in and topics from facilitation meeting
